# Correlation of in vivo imaging to morphomolecular pathology in translational research: challenge accepted

**DOI:** 10.1186/s13550-021-00826-2

**Published:** 2021-08-28

**Authors:** Simone Ballke, Irina Heid, Carolin Mogler, Rickmer Braren, Markus Schwaiger, Wilko Weichert, Katja Steiger

**Affiliations:** 1 School of Medicine, Institute of Pathology, Technical University of Munich, Munich, Germany; 2 School of Medicine, Department of Diagnostic and Interventional Radiology, Klinikum rechts der Isar, Technical University of Munich, Munich, Germany; 3 School of Medicine, Department of Nuclear Medicine, Klinikum rechts der Isar, Technical University of Munich, Munich, Germany

**Keywords:** In vivo imaging, Experimental pathology, Animal models, Translational research

## Abstract

Correlation of in vivo imaging to histomorphological pathology in animal models requires comparative interdisciplinary expertise of different fields of medicine. From the morphological point of view, there is an urgent need to improve histopathological evaluation in animal model-based research to expedite translation into clinical applications. While different other fields of translational science were standardized over the last years, little was done to improve the pipeline of experimental pathology to ensure reproducibility based on pathological expertise in experimental animal models with respect to defined guidelines and classifications. Additionally, longitudinal analyses of preclinical models often use a variety of imaging methods and much more attention should be drawn to enable for proper co-registration of in vivo imaging methods with the ex vivo morphological read-outs. Here we present the development of the Comparative Experimental Pathology (CEP) unit embedded in the Institute of Pathology of the Technical University of Munich during the Collaborative Research Center 824 (CRC824) funding period together with selected approaches of histomorphological techniques for correlation of in vivo imaging to morphomolecular pathology.

Characterization of experimental animal models in translational research ranges from in vivo longitudinal imaging methods to ex vivo histopathological evaluation and downstream molecular analysis. The applicability of results achieved in animal models in general can be problematic and is not always successful [1].

## Need of comparative pathologists

Because of a variety of new technologies during the last years [2, 3], which made it possible to create complex and genetically modulated animal models with the aim to mimic human diseases on a molecular as well as on a morphological level, the number of new animals models, especially mouse models, increases steadily. Besides expected similarities, genetically engineered animal models often show divergences or unexpected alterations resulting in background lesions which easily can falsify results. Therefore, profound knowledge of strain specific background lesions as well as classifications of the occurring pathological alterations in a comparative way is an essential component of translational research. Indeed, there are several examples in the literature where the lack of pathologists from histopathological evaluations of mouse models led to considerable errors in description and diagnosis of histopathological findings [4, 5].

The term “comparative pathology” is defined as pathology of analogous diseases in different species [6]. It can be divided in 3 domains depending on the field of application:

1. *Experimental animal model-based pathology* investigates animal models specifically generated to mimic human diseases [7]
2. *Comparative pathology of spontaneous diseases in domestic and laboratory animals* detects similarities and differences of sick animals to corresponding human diseases to advance diagnostics and therapies in humans [8]
3. *Comparative veterinary pathology* observes and compares diseases of different species which can be biologically highly variable (including humans) [9].

In translational research, comparative pathology is usually used synonymously to experimental animal-based pathology.

Several authors pointed out the looming need of pathologists focusing on comparative aspects for handling the increased use of genetically engineered mice [10–12]. For the last 10 years, the first units and grant programs were dedicated to this topic of research [13, 14]. Still, the number of comparative pathology units in Europe is small, especially when it comes to a direct embedding into an academic pathology department. Some more units have been established in the USA: several centers for comparative pathology are located at veterinary schools, and also some few units are integrated into departments of pathology of medical faculties.

The main reasons for the poor transferability of results in animal model systems to clinical applications with respect to pathology are: 1. missing or incomplete pathologic description/evaluation, 2. lack of current comparative classifications of animal models, and 3. lack in pathologists with appropriate training in exercise of comparative issues [4]. With respect to the correlation of in vivo imaging methods, one could add also a deficiency in the development of protocols and workflows allowing for an appropriate correlation of in vivo imaging techniques with the underlying histomorphological background. In particular, the adequate correlation of images from different in vivo imaging techniques to histological slides can be challenging and requires a high degree of interdisciplinary cooperation. To enable for an evaluation of corresponding planes and the maintenance of 3D-orientation, often different steps of protocol adaptations are needed to achieve satisfying results.

The focus of CRC824 research groups is on oncology. Translational oncological research uses different systems of animal models resulting in different levels of complexity which influence comparability and reproducibility [6]. Table 1 provides an overview of commonly used types of animal models in cancer research.

**Table 1:** Simplified overview of frequently used systems of animal models in translational cancer research

Type			Comment	Main application
Cell line- based	Xenograft (CDX)	Subcutaneous or orthotopic	Also as patient-derived xenograft (PDX)	Cell biology, therapy
	Humanized preclinical models			Immunological research
	Syngraft (CDS)	Subcutaneous or orthotopic	Derived from spontaneously grown, toxically induced or genetically generated endogenous tumors	Cell biology, therapy
Complex, biologically/genetically engineered	Metabolically/toxically induced	Systemic	Mostly based on genetic alterations (targeted induction of a promotor for carcinogen), often organ-specific	Toxicology
	Mutagenesis- screen	Chemical or transposon-mutagenesis	Forward-genetic approaches	Basic cancer research (identification of gene functions)
	Genetically Engineered Models (GEM)	Systemic	Knock-in/knock-out	Translational research (reverse-genetic approaches)
		Organ-specific	Transgenic animals with organ-/cell type-specific promotor	
		Conditional/inducible	Transgenic animals with localized or time-limited genetic alterations	
	Targeted genetically modified animals by genome editing			
	Complex humanized preclinical models			

## The CEP unit

Given the fact that there is a lack of surgical pathologists with background knowledge of animal model pathology and in reverse only very few veterinary pathologists are focusing on translational and imaging issues, it became clear that there is an urgent need bringing the two disciplines together. Besides the appropriate planning and evaluation of animal model-based experiments, a major task of comparative facilities lies in the correct and effective training of pathologists. Additionally, with respect to animal protection and ethics, it must be the aim to decrease animal numbers in research. By planning and evaluating experiments in a scientifically sound way, repetitions and false conclusions can be prevented. The ARRIVE (Animal Research: Reporting of In Vivo Experiments) guidelines as well as the 3R (Replace, Reduce, Refine) principles have to be considered. The majority of animal-based research to date is performed with mice due to their fast reproduction rate and their high availability for genetic engineering methods. There are two main aspects that have to be considered when choosing the mouse as a model organism: 1. characterizing the animal on a morphological and molecular level to ensure that the model used is suitable for the question and 2. correct planning including necropsy and slide preparation as well as consultation of pathologists with expertise in animal model pathology to ensure reproducibility [4, 15].

These circumstances resulted in the establishment of the CEP in 2016, a veterinary pathology unit embedded in the Institute of Pathology at the Technical University of Munich (TUM) consisting of a technical and a scientific part. The CRC824 was the first funding where the CEP unit was involved as a central project (Z-project), meaning a scientific service unit for the research groups included in the CRC824 and focusing on correlation of in vivo imaging techniques to histomorphological evaluation. The CEP rapidly developed and increased within the setting of the CRC824 and became one of the largest comparative pathology facilities in Europe. Started with a basic equipment, it now expanded to a fully equipped and automated histopathological laboratory which enables for processing of the high amount of requests in an appropriate time and way. Its unique feature is the embedding at the Institute of Pathology at the TUM together with a complex network of a broad field of translational research groups focusing on in vivo multimodal molecular imaging. Over the last years, several additional consortial fundings have been added.

**Fig. 1:**
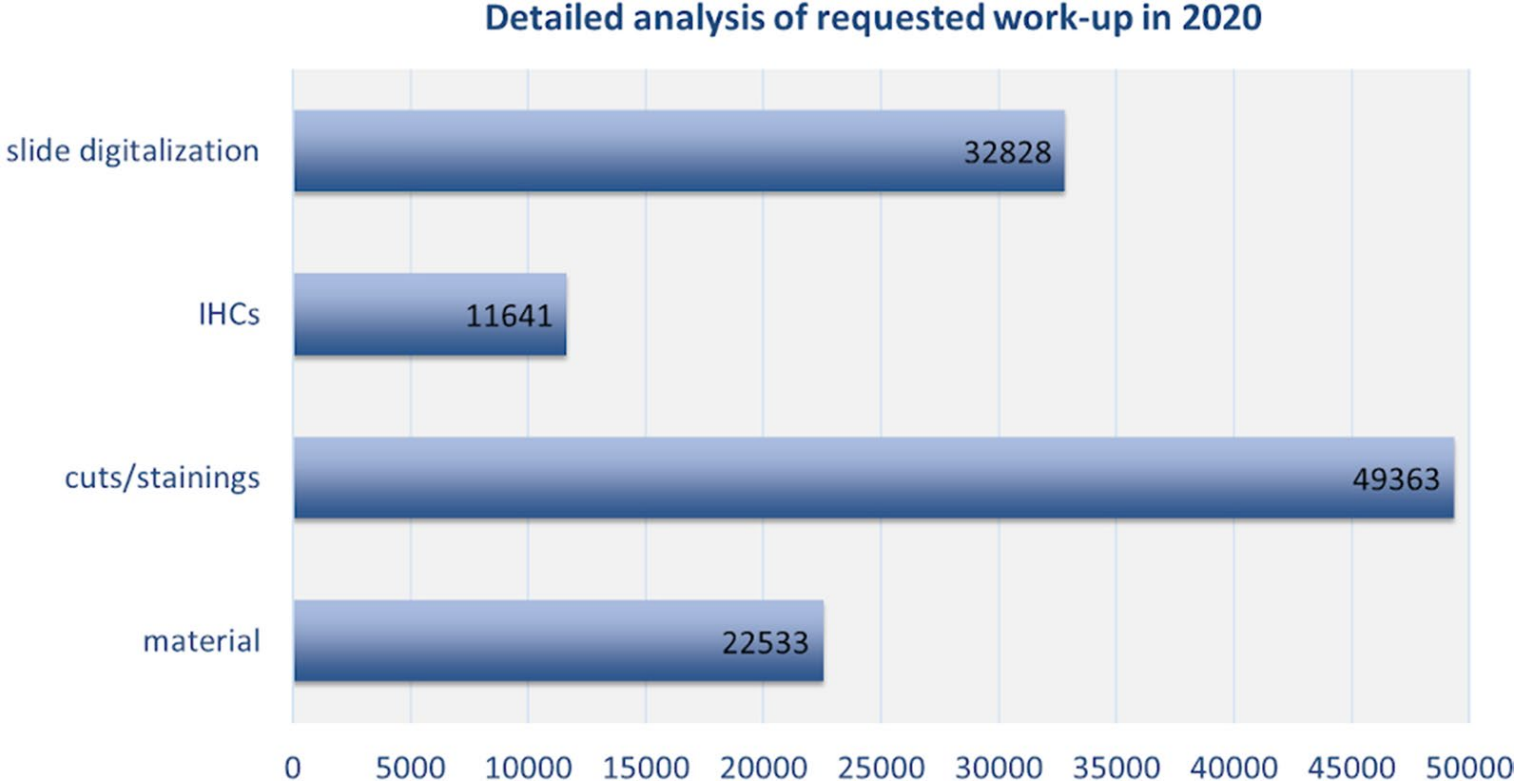
Detailed analysis of the requested work-up in 2020 (material = all incoming material like blocs, empty (unstained) slides and tissues)

Our performance monitoring for the period of 2018 to 2020 showed a total number of processed projects between 500 and 600 per year. Thereof, most projects were mouse studies which accounted for ~ 50% every year (2018: 50.5%, 2019: 48.8% and 2020: 54.0%). Translational projects aiming at comparative evaluation of human tissue specimen also made up a major part of our work: around 40% of projects per year requested comparative human evaluation (2018: 41.6%, 2019: 44.0% and 2020: 39.3%). Rat tissue requests had a lower percentage ranging from 1.7% to 3.2% in the given period. The remaining specimen (around 5% every year) originated from various other species. A detailed analysis of the project requests in 2020 (despite a slight drop due to the Coronavirus pandemic) is shown in Fig. 1. In 2020, in total 521 projects were processed with > 22.500 submissions (ranging from unfixed tissue to already available empty cuts). Nearly 50.000 cuts from formalin-fixed paraffin-embedded (FFPE) and cryo tissue were provided, and in total > 32.000 slides were digitalized. This is not surprising as digitalization in pathology nowadays belongs to standard processes, at least when it comes to research pathology. Technical service was performed in 100% of all projects while scientific support was requested in 75.8%. The technical workup has been performed by 8 technicians employed during the time period analyzed.

For scientific requests, we provided a well-trained team of veterinary pathologists, veterinary pathologists in training and digital pathology expertise. From the surgical pathology site, 9 pathologists (8 consultants, 1 resident) were involved in CEP-projects.

## Imaging and co-localization

As a central project in the CRC824, technical and scientific support of the CEP mainly focused on correlating in vivo imaging to histomorphology.

Basically, co-registrations of in vivo imaging techniques with histomorphological features include exact embedding in the right plane of interest and appropriate labeling of the relevant sites. Besides, it is obligate to ensure appropriate necropsy and fixation of samples. For this reason, pathologists should be involved before starting in vivo imaging or at least before planning of animal necropsy so that the workflow and processing of samples can be adapted in an appropriate way. Table 2 shows the benefit of interdisciplinary cooperation for both sides, researchers and pathologists. As any other research technique, histomorphological sample processing also requires certain defined restriction with respect to time and has to stick to a schedule. It should be avoided to unnecessarily prolong necropsy (because of relatively fast beginning autolysis of tissue) or inadequate or prolonged formalin fixation (because of affecting results of immunohistochemistry). The recommended standard fixation is 10% formalin at room temperature for 48 h. Besides, the ratio of tissue to formalin should be 1:10. Tissue should then be embedded into paraffin immediately without any delay or further change of fixation solutions. In cases where radiation decay is necessary because of the use of radionuclides for imaging, the pathology unit should be contacted in advance to discuss the most appropriate workflow with respect to ensure for the best tissue preservation and subsequent processing for the read-outs needed. It should be avoided to freeze organs for storage and later fix them in formalin for FFPE diagnostics. If necessary, the tissue should be thawed in 10% formalin at 4 °C.

**Table 2:** Challenges and benefits of interdisciplinary cooperation between researchers and pathologists in translational oncology research

Scientific step	Coordinator	Challenges/solutions	Benefit for researchers
**Project management**	Researcher	Definition of question, aim of the study, study and experimental design	
	Pathologist	Experimental design with focus on appropriate mouse models and histopathological evaluation with focus on co-localization	Choosing a suitable animal model for the research question, appropriate/realistic time table
**In vivo imaging**	Researcher	Performance, coordination of procedure and time table in consultation with pathologist	
	Pathologist	Coordination of histopathological tissue processing after in vivo imaging according to time table of researcher	Fast and correct tissue sampling and processing
**Submission of tissue/animals**	Researcher	Inform pathologist before necropsy, planning of necropsy in consultation with pathologist	
	Pathologist	Feedback to researcher about time table, fixation, tissue processing with focus on co-localization, provide appropriate protocols for necropsy	Fast and correct tissue sampling and processing
**Co-localization**			
**- Basic co-localization**	Researcher	Definition of question and aim of co-localization approach	
		Planning of necropsy with focus on special orientation	
	Pathologist	Planning and consulting of necropsy and tissue processing - Flagging by ink - Embedding in the right plane - Consecutive slides	Ensure the correct orientation of tissues for later co-localization
**- Complex co-localization**	Researcher	Definition of question and aim of co-localization approach	
		Planning of necropsy with focus on special orientation	
	Pathologist	Planning/consulting of necropsy/tissue processing with focus on orientation - Removement of tissue with adjacent organ structures - Flagging by ink - Embedding in the right plane - Consecutive slides	Ensure the correct orientation of tissues for later co-localization
**Tissue processing**	Researcher	In consultation with pathologist	
	Pathologist	Time table and coordination with the pathology laboratory (e.g. CEP) - Fixation - Embedding in the right plane, special orientation - Cutting: number and type of slides, consecutive slides - Staining: H&E, additional special stainings	Fast and appropriate tissue processing
		- Immunohistochemistry	Selection of the right target antigen, antibody, protocol
		- Scanning	Simplification of co-localization and co-registration
**Histopathological evaluation**	Researcher	In consultation with pathologist	
	Pathologist	Evaluation of H&E stains, special stainings, immunohistochemistry	Correct histopathological diagnosis because of - Classification of lesions in a comparative way - Standardized vocabulary in description - Knowledge of strain-specific background lesions - Knowledge of differential diagnoses (non-expected alterations)
**Co-registration**	Researcher	Combining in vivo imaging with histopathological findings, in consultation with pathologist	
	Pathologist	Consulting researcher with focus on co-localization of histomorphological findings to in vivo imaging	Use of correct histopathological terms and localization for description of findings
**Summary**	**Interdisciplinary cooperation**		**Accuracy and reproducibility of results**

During the CRC824 funding period, several in vivo–ex vivo co-localization approaches were successfully applied to different issues depending on the complexity of the research question. Basic and quite simple co-localization can be done for easily accessible tumors, like subcutaneous neoplasias, whereas tissue-specific endogenous tumors, like endogenous abdominal neoplasias, are more challenging and require a more complex and difficult workflow for satisfying co-registration results.

**Fig. 2:**
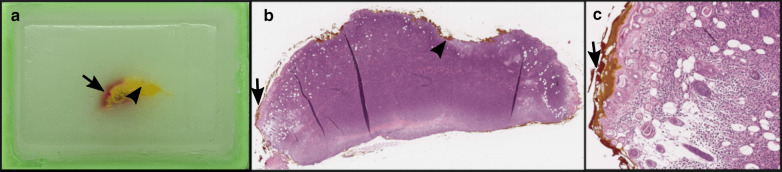
**a** two-colored ink marked paraffin-embedded subcutaneous tumor for basic co-localization (arrow: red ink, arrowhead: yellow ink); **b** H&E stain of **a** (magnification 2 ×); **c** higher magnification (8 ×) of **b**, two-colored ink marked margin of subcutaneous tumor tissue. Although the yellow ink slightly ran into the red ink area, the two colored areas can be well distinguished. Unpublished data

**Fig. 3:**
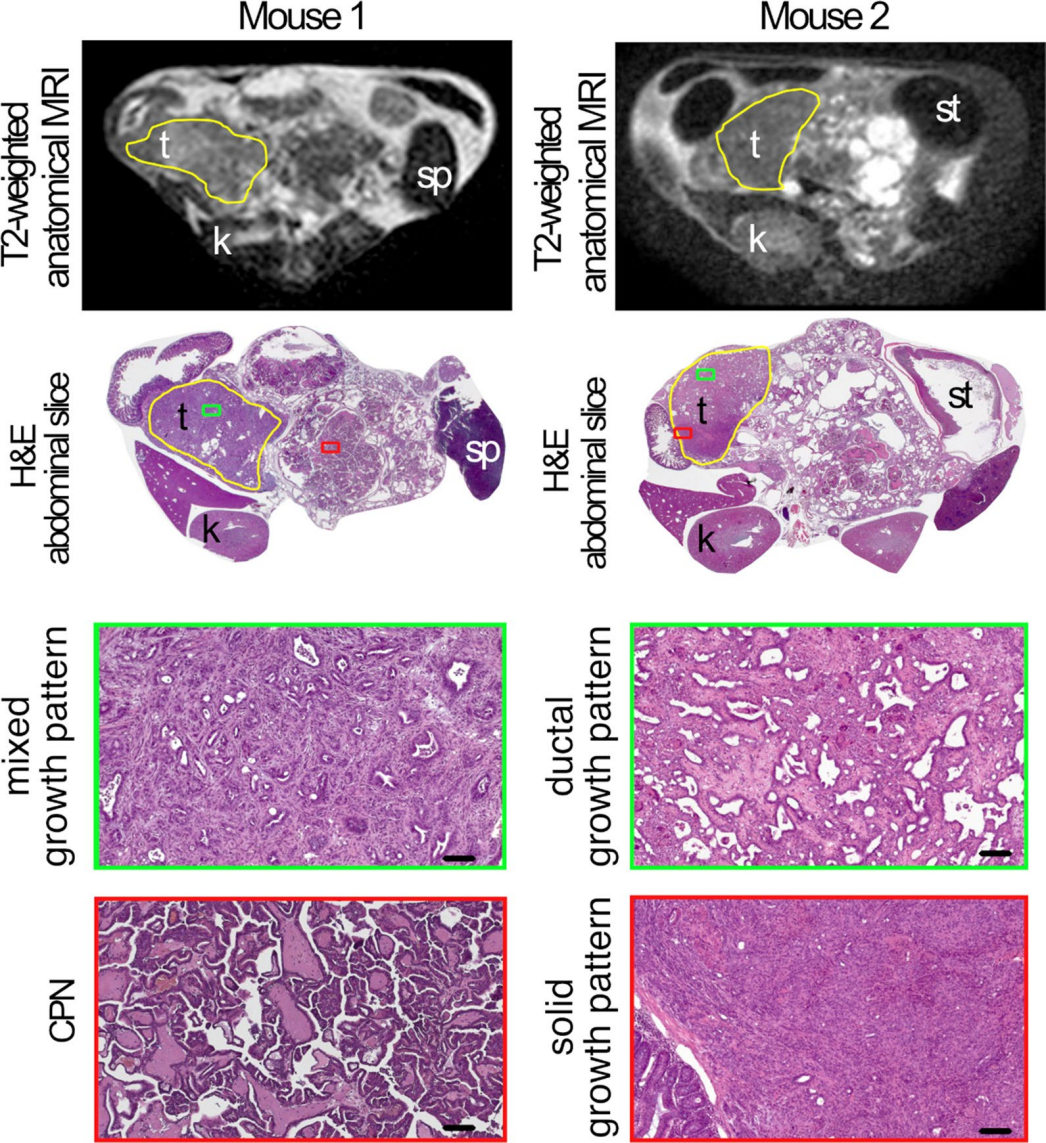
Complex co-registration of anatomical magnetic resonance imaging (MRI) images to histological slides in the transverse plane of abdomen of a mouse (t = tumor, k = kidney, st = stomach, sp = spleen) showing variable tumor growth patterns in different matching regions. Unpublished data

- **Basic co-localization (cell line based xenograft model): solid growing subcutaneous tumors:**
  For example, basic co-localization was done for a murine xenograft model of human myeloid sarcoma aiming at monitoring of human central memory T-cells by Immuno-Positron Emission Tomography (Immuno-PET) imaging [16]. Positron Emission Tomography/Computed Tomography (PET/CT) imaging showed diverse T-cell distribution patterns. Co-localized immunohistochemistry and semiquantitative evaluation of T-cell-infiltration within the tumor was done corresponding to the PET/CT images, which included exact orientation (for example ventral or dorsal), flagging by ink and correct embedding in the right plane (for example downward orientation of the axial cutting site) (Fig. 2). The cutting of consecutive slides is also a useful tool for co-localization. Semiquantitative evaluation of defined markers in predefined regions can be depicted by color codes. Co-localization of imaging and histologic slices can be performed by calculating the sum of total sections, including a variation factor of tissue modification due to the technical procedure.
- **Complex co-localization (genetically modified conditional model): tissue-specific tumors:**
  For tissue-/organ-specific tumors, we developed a workflow for identification of tumor imaging parameters of mouse pancreatic ductal adenocarcinomas (PDACs) and analysis of their applicability for translation to human settings. The study was performed using genetically engineered mouse models that develop an endogenous pancreatic ductal adenocarcinoma [17]. Neoplasms were removed from the abdominal cavity with adjacent organ structures (liver, spleen, gut, kidneys) to prevent change in orientation and to facilitate exact correlation of imaging plane and histology based on additional anatomical landmarks. After proper formalin fixation and paraffin embedding of the whole organ bulk in tissue cassettes, axial histologic slices through mouse abdomen with 4 mm distance could be correlated with in vivo imaging (Fig. 3). Figure 3 demonstrates how pathological examination after exact correlation can complement and improve the readout of the imaging data. By using the technique mentioned above, we were able to correlate areas with different growth patterns (ductal, mixed or solid) or even different types (cystic papillary neoplasm (CPN) vs. PDAC) of neoplasms to certain areas of the invivo imaging slice.
  For complex co-localization of endogenous or orthotopic tumors, we additionally developed different other approaches with respect to the specific research question:

1. For co-localization of detection and monitoring of esophageal cancer severity with capsule optoacoustic endoscopy (COE) in a porcine model, the ex vivo samples were cut along the lumen immediately after imaging to open the esophagus flat and to ensure the precise dissection of the area of interest [18]. Vascular information provided from the novel capsule COE system could be resolved within different esophageal layers and was then confirmed by histology and anti-CD31 immunostaining. Therefore, the exact co-localization of samples as well as an appropriate tissue processing is essential for a correct interpretation of results.
2. Co-localization of the thyroid gland proved to be quite easy to handle when removing the thyroid gland together with the trachea [19]. Together with the trachea, the tumors were excised, fixed in formalin and were available for morphological and immunohistochemical investigations after paraffin embedding. The latter had to ensure the appropriate orientation of the tumor/thyroid/trachea tissue bulk. In questionable cases, ink-based annotation of landmarks or surfaces for orientation of the dehydrated specimen help to ensure appropriate embedding. This approach was used for Galectin-3 targeting in thyroid orthotopic tumors to characterize thyroid cancer [20].
3. Neuroendocrine tumors of the pituitary and the adrenal gland are quite challenging. Exact co-localization of the pituitary gland is only possible if the pituitary remains in the skull. Thus, decalcification is necessary for histomorphological analysis which means that the sample is no longer available for additional methods. Co-localization of adrenals requires the removal of the adrenal with the adjacent kidney, leading to a loss of additional landmarks.
4. For co-localization of lymphomas, one should keep in mind that, in contrast with humans, lymphoma of the mouse occurs multicentrically in lymphnodes [21].
5. Co-localization projects of hepatocellular carcinoma (HCC) nicely showed the difference of levels of complexity between subcutaneous and tissue-specific tumors. Subcutaneous tumors were easily accessible and easy to process for histology after hyperpolarized ^13^C pyruvate magnetic resonance spectroscopy [22]. Processing of endogenous orthotopic HCCs turned out to be more complicated and resulted in a more complex workflow. When processing whole livers or liver lobes for histomorphology analysis, special attention has to be drawn to a proper fixation of the whole tissue bulk. Underfixation, according to our experience, can severely impair the usability of the tissue for pathological examination or immunohistochemistry. If the adequate process of fixation is not known in a laboratory or an imaging unit, in our facility, the CEP team can always be approached to help to develop a proper sampling and fixation setup. In the project mentioned the focus was on taking care to maintain the spatial orientation and relationship of the liver lobes [23, 24].
6. Research requests aiming at correlation of human in vivo imaging with histomorphology of resection specimen included projects working on prostate cancer (unpublished data) and pancreatic ductal adenocarcinoma as well [16, 25].

From the pathologists point of view, for all co-localization issues which include histomorphology and immunohistochemistry, processing of FFPE tissue should always be preferred to cryo sectioning. In cryo sections, cell morphology is not so well maintained and cryo artifacts can interfere with co-localization results. If cryo sectioning is necessary due to a local setup (i.e., availability of a cryotome in the radiation protection area, but lack of a paraffin embedding possibility there), the cryo tissue should be stored unprocessed in liquid nitrogen or embedded in cryo cassettes with an appropriate embedding material and be stored at − 80 °C.

- **Light Sheet Fluorescence Microscopy (LSFM):**

LSFM allows a three-dimensional fluorescence based visualization of whole tissue samples. Two thin counterpropagating sheets of laser light illuminate a thin layer of the sample [26]. Therefore, the tissue has to be rendered transparent to enable the laser light to penetrate the sample (optical tissue clearing). Different clearing protocols have been developed during the last years [27, 28]. By adding LSFM as a mesoscopic imaging tool, it is possible to close the gap between macroscopic and microscopic imaging. Mechanical sectioning of tissue destroys tissue integrity resulting in loss of important information [29]. LSFM allows three-dimensional imaging of intact tissue specimen with micrometer resolution without microtome slicing [26].

Clearing of samples was done using the 3DISCO (3D imaging of solvent-cleared organs) protocol [29]. After imaging with a Light Sheet Fluorescence Microscope (UltraMicroscope (UM) II, LaVision BioTech, Germany), samples were rehydrated by immersing them in descending concentrations of ethanol solutions and finally dehydrated again and embedded in paraffin, maintaining the plane of interest corresponding to UM imaging. Serial sections were done in 50-µm steps to ensure co-localization with UM images [30]. After hematoxylin and eosin (H&E) staining of sections, the slides were co-localized with single images from the UM stack. Accordingly, adrenals of multiple endocrine neoplasia X (MENX)-affected rats which were first labeled with T-lectin for visualizing vessels with LSFM were rehydrated and embedded for co-localization of CD31 and Ki67 after immunohistochemistry to characterize pheochromocytomas as comparable to the pseudohypoxic cluster in humans [31].

## Conclusions

The last decades have shown that genetically engineered animal models are highly adaptable to a great variety of research questions but bring along the difficulty to translate their findings to the human setting. Therefore, the field of comparative pathology together with correlated in vivo imaging became more important. Meanwhile, several authors emphasized the need of pathologists with comparative knowledge focusing on translational research and animal model pathology [4, 10, 32]. As a result, some comparative pathology units developed during the last decade, for example, in Europe the COMparative PATHology platform (COMPATH) at the University of Bern and the Centre for Comparative Pathology at the University of Edinburgh or in the USA the University of Iowa (Comparative Pathology Laboratory) and the Vanderbilt University (Translational Pathology Shared Source—Comparative Pathology). To best of our knowledge, the integrated setup with a comparative pathology unit fully integrated in an Institute of Pathology within medical faculty is still unique in Europe. The CEP unit extended to a fully equipped experimental comparative pathology laboratory for technical and scientific support for research groups with focus on cancer research and therein developed several approaches for correlation of in vivo imaging to histomorphology. Taken together, the embedding of comparative pathology units in university institutes can help to improve and fasten animal model evaluation and can have a major impact on translational research in future (Table 2).

## Abbreviations

CEP: Comparative Experimental Pathology
LSFM: Light sheet fluorescence microscopy
UM: Ultramicroscope
H&E: Hematoxylin–eosin
CRC: Collaborative Research Center
ARRIVE: Animal research: reporting of in vivo experiments
3R: Replace, reduce, refine
FFPE: Formalin-fixed paraffin-embedded
USA: United States of America
PET: Positron emission tomography
CT: Computed tomography
PDAC: Pancreatic ductal adenocarcinoma
MRI: Magnetic resonance imaging
3DISCO: 3D imaging of solvent-cleared organs
MENX: Multiple endocrine neoplasia X
HCC: Hepatocellular carcinoma
COE: Optoacoustic endoscopy

## Declarations

### Availability of data and materials

Please contact the author for data requests.
